# Curbing the Rise of Noncommunicable Diseases in Uganda: Perspectives of Policy Actors

**DOI:** 10.9745/GHSP-D-20-00051

**Published:** 2021-03-31

**Authors:** Ankita Meghani, Charles Ssemugabo, George Pariyo, Adnan A. Hyder, Elizeus Rutebemberwa, Dustin G. Gibson

**Affiliations:** a Johns Hopkins Bloomberg School of Public Health, Baltimore, MD, USA.; b Makerere University School of Public Health, College of Health Science, Kampala, Uganda.; c George Washington University Milken Institute School of Public Health, Washington DC, USA.

## Abstract

**Background:**

Uganda faces a complex policy landscape as it simultaneously addresses infectious diseases and noncommunicable diseases (NCDs). The health system has been overwhelmed by the growing burden of NCDs across all socioeconomic strata. In this study, we sought to understand the policy context around NCDs in Uganda, the roles of actors both within and external to the government, and the factors shaping the development and implementation of NCD policies and programs in Uganda.

**Methods:**

We conducted in-depth interviews with 30 policy actors from the Ugandan Ministry of Health (MOH), nongovernmental organizations, and academia to understand the roles of different actors in the Ugandan NCD space, the programs and policy measures in discussion, and how to bridge any identified gaps. A thematic data analysis was conducted.

**Results:**

All national actors viewed funding constraints as a critical barrier to developing and executing an NCD strategic plan and as a barrier to leading and coordinating NCD prevention and control efforts in Uganda. The crowding of nongovernment actors was found to fragment NCD efforts, particularly due to the weak implementation of a framework for action among NCD actors. Relatedly, limited recruitment of technical experts on NCDs within the MOH was viewed to further diminish the government's role in leading policy and program formulation and implementation. Though recent MOH efforts have aimed at addressing these concerns, some skepticism remains about the government's commitment to increase budgetary allocations for NCDs and to address the technical and human resources gaps needed to achieve NCD policy aims in Uganda.

**Conclusions:**

This study highlights the immediate need to mobilize more resources, reduce fragmented efforts in the NCD space, and prioritize investment in NCD prevention and management in Uganda.

## Abstract (precis)

To respond to the growing burden of noncommunicable diseases (NCDs) in Uganda, technical, managerial, and financial resources must be increased in the Ministry of Health as well as in primary and secondary health care facilities. This investment would help further Uganda's efforts to achieve sustainable development goals and build the government's capacity to meet the increasing needs for NCD services.

## Teaser

**Key Findings:**

- Inadequate government funding for noncommunicable diseases (NCDs) has elevated the role of external partners in shaping the development and implementation of NCD policies and programs in Uganda.
- Limited recruitment of technical experts and managers for NCDs in the Ministry of Health (MOH) has been a barrier to the effective coordination and communication across multiple government and nongovernmental actors in the NCD space.
- Financial, technical, and managerial constraints have contributed to external actors spearheading several NCD program activities with the MOH playing a more supportive role.

**Key Implications:**

- Policy makers and public health practitioners should prioritize building the MOH's financial, managerial, and technical capacity to oversee and lead the development and implementation of NCD programs and policies.
- Government leadership is critical in developing and executing a comprehensive strategic plan for NCDs that all actors can agree upon and adopt as the guiding framework for action.

## INTRODUCTION

The rising prevalence of noncommunicable diseases (NCDs) is a growing concern globally, particularly in low- and middle-income countries, where the burden of disease is transitioning from infectious diseases to NCDs. Every year, NCDs kill 41 million people, and contribute to 74% of the global mortality burden.^1^ NCDs are also the principal causes of morbidity and loss of disability-adjusted life years globally.^2^^,^^3^ Four modifiable behavioral risk factors—unhealthy diet, physical inactivity, excess alcohol consumption, and tobacco use—contribute to roughly 67% of all NCD deaths worldwide.^4^^,^^5^ Globally, nearly two-thirds of NCD-related deaths are caused by cardiovascular disease, cancer, diabetes, and chronic lung diseases.^6^^,^^7^

NCDs are likely to become a major health system challenge in Africa as they are predicted to become the leading cause of death in the region by 2030.^8^ Of the 97,600 deaths in Uganda in 2016, NCDs accounted for 1 in 3.^9^ The estimated risk of mortality from NCDs was 22%, primarily due to cardiovascular disease and cancer, along with underlying risk factors of hypertension, tobacco use, and alcohol.^9^ Across metabolic, environment/occupational, and behavioral risk factors, high blood pressure and high fasting plasma glucose respectively were the sixth and seventh most correlated with death and disability among Ugandans in 2019.^10^ A nationally representative household survey conducted in 2014 further revealed that nearly 80% of Ugandans diagnosed with NCDs were unaware of their own status; for example, only 7.7% of individuals with hypertension were aware of their condition, and among those who had hypertension, 76% were not being treated for their condition.^11^^,^^12^

Population-based interventions, such as mass media campaigns or taxes, may help mitigate the effects of modifiable NCD risk factors. To reduce dietary risk factors, the World Health Organization (WHO) recommends several “best-buy” interventions, such as limiting salt and sugar intake and implementing front-of-pack labelling and taxation on sugar-sweetened beverages.^13^ Implementing mass awareness campaigns and providing counseling during routine primary health care visits about the importance of physical activity for well-being also have been identified as effective NCD interventions.^13^ The successful implementation of these “best buys requires clear policy guidelines and strategies by governments and a concomitant increase in financial and technical investments to support their development.

Locally generated data on NCD risk factors shows a rise in NCDs in Uganda and underscores the need to increase investment in and allocation of resources to NCDs.^11^ The 2015–2020 Ugandan Health Sector Development Plan emphasizes the need to develop national NCD management policy and guidelines, strengthen surveillance systems for NCDs, and expand access to prevention, diagnosis, and treatment services for NCDs, among other related priorities.^14^ The Ugandan government also has expressed commitment to allocate 17% of the total health budget to NCDs and 60% of the NCD budget to prevention services.^14^

At the national level, additional efforts aimed at addressing NCDs include the development of a Multisectoral Health Action Plan for NCDs and several related collaborations with local and global nongovernmental organizations (NGOs) and development partners who support the government in developing trainings, implementing public awareness on NCDs and their risk factors, and strengthening the capacity of the health system to prevent and manage NCDs. In the Ugandan Ministry of Health (MOH), NCD-related policy and program activities have been coordinated by an NCD unit (also known as the NCD Desk at the time of this study). Several promising initiatives are aimed at specific NCDs and their underlying risk factors. These initiatives require identifying unmet needs, understanding the roles of actors both within and outside government working on heterogenous NCD policy and programs, and identifying the factors driving their development and implementation.^14^

It is particularly critical to explore the views of external actors who provide substantial health funding in Uganda (Table 1),^15^ and who may be influential in affecting policy and program development and implementation,^16^ including those for NCDs.^17^ In this context, we aimed to understand the current NCD policy landscape in Uganda, including the role of actors within and external to the government, and then examine the factors shaping the country's development and implementation of NCD policies and programs.

**TABLE 1.** Overview of Key Health Systems and Financial Indicators of Uganda^15^

Indicator	Value	Year
Total population (millions)	42.72	2018
Urban population (% total population)	23.8	2018
Net official development assistance received (constant 2015 US$)	1,976,190,000	2017
Net official development assistance received (% of central government expense)	57.9	2017
Domestic general government health expenditure (% of general government expenditure)	5.14	2016
Domestic general government health expenditure (% of current health expenditure)	16.6	2016
Current health expenditure (% of gross domestic product)	6.16	2016
Out-of-pocket health expenditure (% of total expenditure on health)	40.31	2016
External resources for health (% of total expenditure on health)	40.37	2016

We aimed to understand Uganda's current NCD policy landscape and examine the factors shaping the development and implementation of policies and programs.

## METHODS

### Study Design

Our qualitative study followed an emergent research design using in-depth interviews as our primary data source.^18^ To better understand the NCD policy landscape, we conducted in-depth interviews with 3 types of NCD actors: (1) officials from national government (MOH and Ministry of Finance) with authority to make programmatic and policy decisions for their relevant work areas; (2) researchers from local universities in Uganda working on health policy or familiar with the NCD space; and (3) individuals from NGOs and development partners working in the NCD space. We selected these actors because they made decisions about funding NCD programs; developed, implemented, or evaluated NCD programs in Uganda; or provided technical support to high-level government policy actors. During interviews, we also requested that respondents introduce us to other relevant NCD actors in their networks, including other sectors involved in NCD-related activities, so that we could request interviews with them.

The in-depth interview guide covered NCD programs and policy priorities in Uganda and existing gaps, actors involved in the NCD policy space in Uganda, and factors influencing the development or implementation of NCD policies and program (Supplement). To inform the development of a semistructured, in-depth interview guide, we read key policy documents pertaining to the national government's strategic plans for health and health financing. These documents included the 2015–2020 Uganda Health Sector Development Plan,^14^ the 2016 Health Financing Strategy,^19^ and the 2019–2020 National Budgetary Framework,^20^ as well as other documents about preventive and curative services for NCDs in Uganda.^21^^–^^23^ We piloted the in-depth interview guide with 4 health policy researchers working in the NCD space who were not selected to participate in the study.

### Data Collection

In-depth interviews were conducted from February to July 2018 by 3 research staff of the Makerere University School of Public Health who were trained qualitative researchers with several years of experience conducting policy-level interviews in Uganda. Prior to data collection, interviewers completed a 2-day training session on research methods for qualitative data collection and principles of research ethics. Written informed consent was obtained from each participant prior to conducting the interview. All interviews were conducted in English and audio recorded. Interviews lasted between 45 minutes and 1 hour. Study staff transcribed all audio-recorded interviews verbatim immediately following each interview.

During the interview process, the study team met weekly to reflect on the interviews, identify potential problems and areas of improvement, revise the interview guide, and discuss the focus of future interviews. This iterative process aligned with the emergent nature of our study design, as analysis of early interviews informed the selection of topics for subsequent interviews. Throughout this process, we triangulated responses from different NCD actors to develop a comprehensive understanding of the policy context, to identify any areas of consensus and divergence, and to examine factors affecting NCD policy/program development and implementation in Uganda.

All participants we identified agreed to participate in an in-depth interview. Overall, we conducted 30 in-depth interviews with NCD actors from the MOH, Ministry of Finance, academia, and local and international NGOs, as well as development partners working in the NCD space in Uganda (Table 2).

**TABLE 2.** Policy Actors in Uganda Involved With Noncommunicable Diseases Who Participated in In-depth Interviews

Type of Respondent	Number	Corresponding Identification Number
Academics	7	A1-A7
National government officials (ministries of health and finance)	10	G1-G10
Members of civil society organizations, nongovernmental organizations, and semi-autonomous agencies and development partners	13	C1-C13
Total	30	

### Analysis

A thematic analysis was used to analyze the in-depth interview data, primarily using deductive coding.^24^ We grouped textual data according to elements of the policy analysis triangle^25^ (context, actors, process), and drew from the 4-stage policy heuristic (problem identification, policy formulation, policy implementation, and policy evaluation). Actors were identified as specific individuals, groups, or organizations currently or potentially involved in the NCD space in Uganda, including national-level actors from government, academia, and international development partners. Context was viewed as situational, cultural, and international factors that could influence policy or program perspectives around NCDs. Processes were the steps involved in different stages of policy development, from problem identification to policy formulation, implementation, and evaluation. Focusing on these elements provided a conceptualization of the existing landscape around NCD programs and policies in Uganda. We also inductively coded data not captured through our deductive approach to identify factors shaping the development or implementation of NCD policies and programs in Uganda.^26^

Initially, 2 co-authors analyzed the first set of 6 transcripts to review and reconcile the codes, which then informed the final codebook. The codes were organized into 3 main categories: context, actors, and process. The process category was further divided into problem identification, policy formulation, policy implementation, and policy evaluation, for which additional codes emerged related to the factors shaping these processes (e.g., technical, financial, and managerial). Codes were analyzed for each category, and memos were written to summarize emerging themes. Atlas Ti.8.0 was used to manage the textual data and support coding.

### Ethical Approval

We received ethical approval from the institutional review boards at Makerere University School of Public Health (protocol 526) and the Uganda National Council of Science and Technology (registration number SS 4477). The institutional review board at Johns Hopkins University Bloomberg School of Public Health provided an institutional review board exemption for this study.

## RESULTS

### The Current Landscape: Actors and Platforms for Engagement in the NCD Policy Space

#### Overview of Actors

The NCD policy space in Uganda is diverse and includes a multitude of government, nongovernment, and development actors involved in developing and implementing various programs and policies across the country. Respondents identified several departments, divisions, and units within the MOH that are involved in NCD programming and policy making, such as the Division of Mental Health and Substance Abuse, Division of Tobacco and Alcohol, Policy and Planning Unit, Health Services Directorate, and Public Health and Preventive Unit. However, all respondents agreed that the NCD Desk, now the NCD Department, is a critical coordinating body because of its perceived convening power to bring together actors within and outside the government.

In contrast, respondents viewed external actors as largely providing the MOH with technical assistance in developing policies, strategies, plans, and guidelines for NCDs, and in many instances, supporting the implementation of pilot projects and programs. In particular, semi-autonomous government agencies, such as the Uganda Cancer Institute and Uganda Heart Institute, were seen as advisers to the MOH on matters related to their disease specialty. These 2 institutes and WHO were identified as having close partnerships with NGOs, such as a research partnership with the Ugandan Initiative for NCDs to support implementation of disease prevention and management programs at the community level.^27^ Respondents highlighted several projects aimed at improving management of NCD services among lower-tier health centers, training health workers on NCDs, and implementing sensitization campaigns to increase NCD awareness among communities, district councils, and parliamentarians.

In addition to being program implementers, NGOs also were seen as NCD advocates who encourage the development of government programs and policies that expand access to preventative care and treatment for NCDs. As part of this role, NGO actors reflected on their past engagements with policy makers, which included hosting NCD Awareness Days in the parliamentary offices and funding visits for parliamentarians to learn about the types of policies and programs being implemented to address NCDs in other countries.

#### Platform for Engagement

Respondents identified multiple platforms that facilitated engagement between government and external partners, such as steering committees for specific NCDs and, more recently, a multisectoral committee comprised of representatives from different government ministries (e.g., agriculture, transport, education, health) and external stakeholders. The NCD Parliamentarian Forum was seen as a platform to engage with parliamentarians, to understand their views on NCDs and related policies, and to share information about addressing NCDs for the better health of their constituents.

The NCD Technical Working Group, which is housed in the MOH but coordinated by the NCD Desk, was seen as an important knowledge-sharing and policy-making forum where both programmatic and research activities could be disseminated and shared with the MOH and its external partners, such as researchers, civil society, and development partners. Respondents felt this forum facilitated discussion about the latest evidence on NCDs in Uganda that could inform MOH guidelines, policies, and programs for NCD prevention and care.

### Factors Affecting the Development and Implementation of NCD-Related Policies

Our interviews with policy actors suggested that the level of financial, managerial, and technical resources available to them affected what roles they could play in developing and implementing policies for NCDs in Uganda (Table 3).

**TABLE 3.** Financial, Managerial, and Technical Barriers to the Noncommunicable Disease Policy and Program processes in Uganda

	Financial (control and allocation of funds to NCD-related activities; overall funding in the health sector)	Managerial (ability to lead on policy issues, coordinate efforts among government & partners, oversee implementation)	Technical (ability to produce, analyze, interpret, and influence policy/programming)
**Policy/Program Formulation**	NCD Department (previously Desk) has limited ability to mobilize resources, including human resources, necessary for the finalization and execution of the NCD Strategic Plan Government unable to commission studies to fill NCD knowledge gaps	Inadequate recruitment of techno-managerial human resources limits the NCD Department's ability to manage and coordinate processes within the Ministry of Health to move the agenda for NCDs forward Reliance on external partners to address gaps in coordinating efforts around policy/program development	Primary study on NCDs done in 2014 was donor-funded/partner-led; need more evidence; limited role of government in driving research efforts Reliance on external partners for technical assistance and policy formulation based on evidence they generate
**Policy/Program Implementation & Monitoring**	Limited funding for implementing established guidelines Lack of public sector funding has elevated the role of partners and nongovernment organizations that are leading implementation Due to limited funding, the government's role is limited to supervising activities funded and implemented by external partners	Program implementation activities are largely done by external partners with minimal government oversight Inadequate government oversight and coordination results in duplication of programs implemented by different actors Fragmentation of efforts results in external partners working in silos	
**Cross-cutting Issues**	Inertia to change existing funding practices, particularly if cuts are made to other disease areas Absorptive capacity of the government may be limited after partner-run programs end External actors provide significant financial and techno-managerial support in policy/program formulation and implementation processes		

Note: Table structure adapted from Khan (2018).

Abbreviation: NCD, noncommunicable disease.

#### Financial Resources

Policy actors felt that insufficient funds were allocated for NCD-related activities by the government and that this deficiency affected the formulation, implementation, and subsequent monitoring and evaluation of NCD programs and policies. The 2015–20 Uganda Health Sector Development Plan describes the importance of providing a comprehensive package of essential health services encompassing NCD prevention, control, and management. In this plan, 17% of the health budget is allocated to NCDs, and of that, 60% is directed toward prevention activities. However, a funding gap of 29% was identified as a challenge for achieving Uganda's health agenda.^14^ According to the National Budget Framework 2019–20, the health sector received 8.9% of the total government budget.^20^ Both government and external actors reported that the proportion of this budget dedicated to NCDs was insufficient, particularly given the need to develop an NCD infrastructure that includes primary and specialized health care.

Both government and external actors reported that the proportion of the health budget dedicated to NCDs was insufficient, particularly given the need to develop an NCD infrastructure that includes primary and specialized health care.

**Policy Formulation**. The most recent National Health Policy of 2010 recognizes the growing burden of NCDs. However, respondents felt that policy discourse was not enough. Respondents universally expressed the need for an overall strategic plan for NCDs to provide both government and external actors with a cohesive and coordinated blueprint for the prevention and management of NCDs in Uganda. ^28^ Such efforts require additional funding, as a government official noted:

*When you go to the National Health Policy, you will find that NCDs are prioritized in the Health Sector Development Planning document, but this unfortunately does not lead to increased funding for NCDs.*—Government official, G5

Respondents also felt that the lack of financial resources available to the NCD Desk constrained its ability to lead and coordinate the efforts necessary for developing the NCD strategic plan.

*Things need money, the staff and the implementation at least. They [MOH] have started to talk about it … but still, in terms of the money, it is still lacking. It is a very scanty program, but they had promised to upgrade the department.*— Civil society organization member, C7

Specifically, respondents felt that limited funding resulted in inadequate recruitment of technical and programmatic staff to manage and coordinate processes within the MOH to move the agenda for NCDs forward. Consequently, external partners, such as WHO, filled the gaps. These external partners were viewed as playing pivotal roles in helping to develop guidelines and plans for different NCD areas and in collaborating with government and civil society organizations to develop clinical guidelines for managing NCD care at different levels of the health system.

At the time of our interviews, there were discussions within the MOH about converting the NCD Desk into the NCD Department, which became official in July 2019. Many respondents expressed that as members of a full-fledged government department, NCD program officers would have the necessary funding, power, and decision-making ability to facilitate the development, implementation, and expansion of appropriate NCD programs and policies. Furthermore, the graduation from a desk to a department would mean more funding, which respondents felt would reflect the MOH's prioritization of NCDs. As a government official noted:

*We have elevated the NCD Desk to a Department. That shows, first of all, that the Ministry [of Health] is determined to implement that strategy.* —Government official, G7

Aside from slowing the development of the NCD strategic plan, some respondents indicated that a lack of financial resources limited the government's ability to commission new studies to understand the prevalence and distribution of NCDs and their risk factors across the population. For example, respondents felt that studies were needed to assess NCD risk factors among young people, such as childhood obesity. Respondents also felt that financial constraints limited the government's ability to formulate appropriate NCD policies and guidelines, leaving such efforts to actors with access to financial resources, namely development partners or NGOs.

**Implementation and Monitoring of Policies and Programs**. Respondents felt that the lack of government funding for NCDs elevated the role of external actors in the NCD space in Uganda. As such, WHO, civil society actors, and NGOs played more active roles in the development and implementation of different programs to address their own mandates for cancer, heart disease, tobacco control, and so on. One MOH respondent described the diminishing role of the MOH as follows:

*The Ministry, because of the little funding we have, most of the programs are run by partners. We just come, maybe to monitor and to support those programs* …—Government official, G5

Respondents felt that the lack of government funding for NCDs elevated the role of external actors in the NCD space in Uganda.

Though respondents from the MOH felt that the lack of funding did not limit their ability to discuss government priorities with donors, they still felt that funders tended to be more influential in deciding how programs should be implemented. They described how funders' priorities and plans needed to be followed most of the time:

*Mostly, the funders' influence how the things should be done, because if they come up with their plan of implementation, you have to follow it if you are to implement this kind of project or program. You must follow their priorities and their plans most of the time. If the Ministry is … going to implement, we also have to discuss with them our mandate and our priorities, so that we all benefit. … They come with their priorities. We also have ours. So, sometimes we have to discuss and say, “Let us maybe do this and this,” depending on what we want to implement* … —Government official 3

Respondents felt that planning, programming, and implementation would be different if the MOH had more funding. As such, senior leaders of civil society organizations operating in the NCD space felt that funding constraints shifted the balance of power toward nongovernment actors and thus limited the government's ability to set priorities and the agenda. Appropriate funding allocation by local governments to support the implementation of national NCD programs and policies was highlighted as a critical factor, as indicated by one senior government official:

*The local governments have to deliver. The local governments have to make sure that the facilities are there to diagnose and treat the patients, so we need to see that more money is earmarked, for example, to provide the drugs that are critical or vital in the management of NCDs.*

#### Managerial and Technical Expertise

Respondents felt that building the technical and management capacity of the MOH to lead and coordinate the work around NCDs was critical for (1) coordinating different NCD program activities conducted by different actors; (2) strengthening existing policy processes, such as facilitating workshops with stakeholders to develop guidelines and policies for different NCD programmatic areas; and (3) improving the implementation of NCD guidelines, including the NCD component of the Health Sector Development Plan. Respondents also felt that hiring staff with technological and managerial skills for the NCD Desk would increase its presence and awareness of NCD-related issues within the government.

Respondents felt MOH technical and management capacity needed to be strengthened to lead and coordinate the work around NCDs.

First, several respondents, including actors from the government, civil society, and NGOs, expressed frustration over the perceived lack of leadership from the MOH in coordinating this effort. Specifically, respondents found the managerial and technical capacity of the MOH to be inadequate and incomplete. Respondents both within and outside the government felt that involvement from multiple actors without adequate coordination by the MOH created silos and fragmentation in policy and program implementation efforts in the country. Without clear agreement on how best to manage NCD prevention and care in Uganda, respondents felt resources were not being used effectively. One respondent described the lack of communication as follows:

*We could be strategic in leveraging resources and getting the most out of our investment, because now I don't know much about what is happening in other areas of NCDs, other than what I am leading* … —Civil society organization member, C4

Lack of communication from the MOH often resulted in information about new policies and programs reaching only those who attended meetings and not reaching policy implementers or beneficiaries who, according to a civil society organization member, “may not even know what is in it.”

Second, in a context where most actors operate outside of the MOH, senior policy actors within the MOH felt that programs were being developed and implemented to meet the funder's mandate rather than to address an overarching strategic plan. One government official put it this way:

*There are different people doing different things. It just depends on where someone gets their money.*—Government official, G1

Another respondent echoed this sentiment, further highlighting how the absence of technical and managerial leadership, including internal agreement on NCD strategy and priorities, diminished the government's ability to control and influence the implementation of NCD programs.

Third, respondents felt that inadequate recruitment of managers and technical experts within the MOH limited the government's technical role in policy and program formulation. Some respondents felt that the government did not actively drive the research agenda on NCDs, leaving such effort to external partners, who also produced the evidence. At the time of this study, other than the 2014 NCD survey conducted by the MOH with technical support and funding from WHO,^11^ respondents were not aware of any other government-commissioned study. Although they felt that this report's findings could form a strong basis for developing an NCD strategic plan, they also emphasized that additional areas required more research, such as identifying risk factors affecting adolescent health and predispositions to NCDs.

In sum, respondents universally emphasized the important managerial and technical roles of the NCD Desk while also recognizing that mobilizing actors is a particularly large challenge due to understaffing and inadequate financing.

#### Cross-cutting Issues

The inertia to change existing funding practices and the limited absorptive capacity of the government were identified as cross-cutting issues affecting the availability of financial resources and appropriate staffing of technical experts and managers by the MOH. Respondents felt that the declining government expenditure on health reflected a lack of prioritization of the health sector. Without an increase in overall funding for the health sector, respondents felt areas like NCDs would remain underfunded.

According to one academic interviewed, changing budget allocations within the MOH from areas that historically received the “lion's share of funding” to other underfunded areas was viewed as particularly challenging for 3 reasons. First, respondents recognized that reallocating funds within the limited resources of the current budget would result in funding cuts to existing areas that are already underfunded:

*If they [MOH] are to move money from infectious disease control to put it in NCDs, it will mean that they have managed and controlled infectious disease to a level where they can go on to another disease area, but they haven't done that yet.* —Academic, A1

Second, some respondents acknowledged the powerful role of lobbyists in Uganda who maintain the status quo and protect NGO interests. One respondent from the MOH noted that compared to issues like HIV and family planning, NCD-related issues receive less government funding and less interest from the donor community to fund civil society organizations and other networks. Third, a couple of respondents felt that funding for NCDs would remain low as long as politicians and elites in power do not directly experience NCD consequences. As one respondent explained, access to health insurance shields politicians from the adverse consequences of NCDs, and funding for NCDs thus remains a barrier.

## DISCUSSION

In this study, we aimed to understand the policy context for NCDs in Uganda by eliciting the views and experiences of a wide range of stakeholders within and external to the MOH. In addition, we sought to identify the factors influencing the development and implementation of policies and programs for NCDs within Uganda. From the in-depth interviews, it became clear that the NCD space has multiple actors, such as NGOs, alliances for specific NCDs, and development partners, who play major roles in shaping the development and implementation of NCD policies and programs. Although these activities by external partners serve as valuable stopgap measures, respondents noted several challenges facing the government.

First, many respondents expressed that the allocation of financial resources toward the development and implementation of existing NCD programs remains a challenge. Though the overall health budget has increased in recent years in Uganda, resources available for NCD policy implementation have not met needs.^17^ A recent evaluation examining priority settings for NCDs in Uganda similarly noted delays or failure to implement relevant NCD priorities due to insufficient resources and stated a need for leadership and greater accountability to support these processes.^16^ The mismatch between the disease burden of and response to NCDs is not unique to Uganda. It has also been observed in other countries in sub-Saharan African, where efforts to address NCD prevention and burden has been inadequate.^29^^–^^31^ Additional funds will be required to target the changing trends in NCDs and their underlying risk factors. For example, tobacco use among adults has declined, but incidences of obesity and high blood pressure have increased.^9^ Expanding annual health budgets and earmarking funds for NCDs will be critical. However, traditional funding approaches (e.g., domestic tax revenues), though a common source of health funding in low- and middle-income countries, have been described as inadequate to close the funding gap required for NCDs.^32^ In this context of limited resources, the role of innovative financing mechanisms (e.g., pooled funding) to mobilize alternative financing strategies to support the prevention and management of NCDs may be useful to consider.^33^

Second, the limited recruitment of managerial and technical expertise for the NCD Desk (at the time of the study) was viewed as a barrier to the coordination and communication across actors within the NCD space. Platforms such as the NCD Technical Working Group and Parliamentary Forum on NCDs allow different stakeholders to engage with one another. Yet, external partners often lead policy and program development, with the MOH playing more of a supportive role. This situation is not unique to Uganda. Studies in other low- and middle-income settings have similarly noted diminished roles of local policy actors in the policy process, particularly when they lack sufficient “control” of financial and technical resources^34^^,^^35^ and when collaboration and coordination between the government and partners is weak.^35^^,^^36^

Respondents felt the presence of multiple external actors led to a perceived vacuum in government leadership and alluded to a need for stronger government leadership in developing a comprehensive strategic plan that both government and external actors can agree upon and adopt as the guiding framework for action. If the activities of actors in the NCD space are well-coordinated and channeled to implement a clear strategic plan, then the presence of different actors could be advantageous. Strengthening platforms such as the Health Policy Advisory Committee; conducting regular joint reviews between the government, NGOs, and funders; and documenting priority areas for action may be mechanisms for strengthening the government's stewardship role in a multi-partner and multi-stakeholder environment for NCDs in Uganda. Developing a policy vision and strategy, building a coalition of relevant actors in the space, and creating accountability for action have been identified as additional ways to strengthen stewardship.^37^

Respondents alluded to a need for stronger government leadership in developing a comprehensive strategic plan that both is both government and external actors agree upon and adopt as the guiding framework for action.

Third, respondents raised concerns about the government's absorptive capacity and ability to take on financial, managerial, and technical responsibilities for overseeing various NCD programs initiated by external partners. In 2015, a benchmarking exercise conducted by the East Africa NCD Alliance Initiative described a need for greater alignment between development partners and national governments, more robust implementation frameworks, and stronger health system capacity to manage NCDs and address the growing burden of NCDs in low- and middle-income countries, including Uganda.^38^ A recent assessment in Uganda found that only 34% to 48% of the country's public health facilities offer services for diabetes, cardiovascular disease, and chronic respiratory diseases.^14^

This study highlights the immediate need to mobilize more resources, reduce fragmented efforts in the NCD space, and prioritize investment in NCD prevention and management in Uganda. Since this study was completed, the MOH elevated the NCD Desk to a full NCD Department headed by a commissioner. The expansion is expected to increase funding and staffing of human resources dedicated to the prevention and control of NCDs. The NCD Department at the MOH, in partnership with health partners, has begun training health workers, including community health workers in NCD prevention and control, at the facility and community levels. The MOH also has supplied health facilities with new blood pressure machines and weighing scales to improve NCD control and management. There have been discussions about adding new NCD drugs to Uganda's list of essential medicines, which would facilitate their procurement by the National Medical Stores and increase their distribution to lower-level facilities. In addition, the MOH engaged with the pharmaceutical industry through public and private partnerships to increase access to NCD drugs at lower costs.

The MOH elevated the NCD Desk to a department, begun health worker training in NCD prevention and control, and provided new equipment and supplies to facilities.

Despite these achievements, a strong policy is still needed to guide prevention, screening, treatment, and management of NCDs. In August 2019, the MOH launched the Non-Communicable Diseases and Injuries Commission with the goal of reframing NCD and injury care in Uganda. The Commission has been tasked with collecting, analyzing, and reporting information to demonstrate the national burden related to NCDs, assess the health system's readiness, and propose mechanisms to strengthen health system capacity.^39^ Proceedings from the Commission are expected to bring greater attention to the policy and funding needs to strengthen NCD prevention and management in Uganda.

### Limitations

Our study has several limitations. First, despite trying to interview a broad range of actors from different organizations, we were unable to interview some key policy actors, such as parliamentarians and actors from other sectors outside of health whose insights would have contributed to shaping our narrative. As a result, we captured a subset of views that may not represent all views on these issues. Second, because we conducted interviews with national-level actors, we were unable to completely explore factors that affect policy implementation at the sub-national levels. Third, conducting interviews with policy actors often requires insider access to information and resources, as well as trust. Although the study team consisted of Makerere University School of Public Health faculty, it is possible that respondents might not have been fully transparent with the interviewer, thus preventing a more nuanced narrative.^40^ Relatedly, differences arising from the investigator's ability to establish and maintain rapport with respondents, their interviewing styles, as well as the time respondents could dedicate to the interview, affected the type of data collected and the depth of discussions. Finally, our work was specific to Uganda, and thus we recognize that our findings may not be widely generalizable. However, we anticipate that some of our findings may be relevant to governments and partners working in other countries that are trying to address the growing double burden of disease in the face of limited resources.

## CONCLUSIONS

Awareness about the importance of addressing NCDs is increasing in Uganda, as reflected in Uganda's policy discourse, the development of an NCD Department within the MOH, and efforts to engage with partners, researchers, and other stakeholders, such as the NCD Technical Working Group. Funding constraints continue to hamper the government's ability to lead in the NCD space and contribute to an imbalance, with external actors spearheading several NCD program activities in the country. Despite recent improvements, respondents remain skeptical about the government's commitment to adequately increase budgetary allocations to meet NCD-related needs and to invest in strengthening health system capacity by addressing technical and human resources constraints.

## Supplementary Material

20-00051-Meghani-Supplement.pdf

## Notes

**Peer Reviewed**
