# Supplementary material for: Curbing the Rise of Noncommunicable Diseases in Uganda: Perspectives of Policy Actors
Source: Glob Health Sci Pract. 2021 Mar 31;9(1):149–59. doi: 10.9745/GHSP-D-20-00051 (PMC8087435; doi:10.9745/GHSP-D-20-00051)
Supplement: 20-00051-Meghani-Supplement.pdf [file 20-00051-Meghani-Supplement.pdf]

Supplement to: Meghani A, Ssemugabo C, Pariyo G, Hyder AA, Rutebemberwa E, Gibson DG. Curbing the rise of noncommunicable diseases in Uganda: perspectives of policy actors. *Glob Health Sci Pract*. 2021;9(1). https://doi.org/10.9745/GHSP-D-20-00051

CURRENT STATE OF NONCOMMUNICABLE DISEASE PROGRAMS & POLICIES IN UGANDA

KEY INFORMANT INTERVIEW GUIDE

Date of interview:

Name of interviewer:

Start time of interview:

End time of interview:

INTRODUCTION

Thank you for taking the time to speak with us. The aim of our study is to learn about the state of non-communicable diseases (NCDs) in Uganda, including current programs and policies for NCDs implemented by the government, and other actors. In addition, we would like to learn about the factors that influence the development and implementation of new programs and policies related to NCDs.

Interviewer: Request informed consent, and permission to turn on audio recorder

BACKGROUND CHARACTERISTICS OF THE INFORMANT

Interviewer: Document/verify the following information in a separate document

a) Participant name, current position, organization/affiliation
b) Contact information
c) Length of employment in current position

INTERVIEW GUIDE

1. Could you please describe your current role(s) & responsibilities in this position?
    a. Any work relating to NCDs?

2. Are you familiar with the programs and policies relating to NCDs that are currently in place in Uganda?
    a. If not, is there someone you suggest I speak with? *[End interview]*
    b. If yes, continue the interview. Move to Question #3

3. What are the existing programs/policies for NCDs in the country?
    a. What has been the focus of these programs/policies?
    b. Who has led these initiatives? What is the role of government vs partners (e.g., NGOs and civil society)?
    c. In your opinion, have the programs/policies been successful? Are there any limitations?

4. What are current government's priorities when it comes to NCDs? Are there certain NCDs (or services for NCDs) that the government has been prioritizing?

Supplement to: Meghani A, Ssemugabo C, Pariyo G, Hyder AA, Rutebemberwa E, Gibson DG. Curbing the rise of noncommunicable diseases in Uganda: perspectives of policy actors. *Glob Health Sci Pract*. 2021;9(1). https://doi.org/10.9745/GHSP-D-20-00051

5. Can you tell us more about NCD program/policy development process?
    a. Who has been leading the process? Who has been supporting the process?
    b. What has worked well?
        i. Probe: Any facilitators?
    c. What hasn't worked as well?
        i. Probe: Any barriers?

    Probes: National NCD policy, Multisectoral Action Plan for NCDs

6. Who are the actors that the government is closely working with on NCD programs/policies?
    a. How have these actors influenced the development of NCD programs/policies?
    b. How have they influenced program/ policy implementation?
    c. Which actors have been influential?
        i. Probe: Local NGOs, international organizations

7. What are the current NCD programs funded by partners? Are there plans to transfer the programs to the government? What role is the government currently playing in this respect?

8. Now, based on your perspective - what types of programs/policies addressing NCDs is most needed? *Note: allow the respond to first list out barriers; then probe for clarification in the following areas:*

9. Is there anything else you would like to share with us?

CLOSING

- Request for any documents related to NCD polices and program in Uganda.
- Request for names and the contact information of other potential interviewees

Thank you for your time, and for participating in this study. We appreciate your insights, as they will be very helpful in helping us understand the current policy and program landscape around NCDs in Uganda.
